# Biomolecular phase separation in stress granule assembly and virus infection: Stress granule and virus infection

**DOI:** 10.3724/abbs.2023117

**Published:** 2023-07-03

**Authors:** Yi Liu, Zhiying Yao, Guiwei Lian, Peiguo Yang

**Affiliations:** Westlake Laboratory of Life Sciences and Biomedicine School of Life Sciences Westlake University Hangzhou 310030 China

**Keywords:** liquid-liquid phase separation, stress granule, viral infection, anti-viral response, biomolecular condensate

## Abstract

Liquid-liquid phase separation (LLPS) has emerged as a crucial mechanism for cellular compartmentalization. One prominent example of this is the stress granule. Found in various types of cells, stress granule is a biomolecular condensate formed through phase separation. It comprises numerous RNA and RNA-binding proteins. Over the past decades, substantial knowledge has been gained about the composition and dynamics of stress granules. SGs can regulate various signaling pathways and have been associated with numerous human diseases, such as neurodegenerative diseases, cancer, and infectious diseases. The threat of viral infections continues to loom over society. Both DNA and RNA viruses depend on host cells for replication. Intriguingly, many stages of the viral life cycle are closely tied to RNA metabolism in human cells. The field of biomolecular condensates has rapidly advanced in recent times. In this context, we aim to summarize research on stress granules and their link to viral infections. Notably, stress granules triggered by viral infections behave differently from the canonical stress granules triggered by sodium arsenite (SA) and heat shock. Studying stress granules in the context of viral infections could offer a valuable platform to link viral replication processes and host anti-viral responses. A deeper understanding of these biological processes could pave the way for innovative interventions and treatments for viral infectious diseases. They could potentially bridge the gap between basic biological processes and interactions between viruses and their hosts.

## A Brief History of Liquid-Liquid Phase Separation (LLPS) and the General Principles of Phase Separation in Stress Granule Formation

### A brief history of stress granule research

To our knowledge, the term “stress granule (SG)” was first used in a 1988 paper characterizing the heat shock protein granules formed in chicken embryo fibroblasts [1]. In this study, SGs were described as electron-dense, membrane-less, cytoplasmic structures that generally do not exist in healthy cells. Another technical definition of SGs was provided by Paul Anderson’s group in a 1999 paper: SGs are “operationally defined as the cytoplasmic foci at which untranslated mRNAs accumulate in response to stress” [2]. Later studies showed that SGs are widely formed in various species, from single-cell yeast [3] to worm [4], fly [5], plants [6], and animal models [7].

Stress granules comprise ribonucleoprotein complexes formed under various internal and external environmental stimuli, mainly in the cytoplasm. The so-called nuclear stress granules contain the HSF1 transcription factor, primarily formed under heat shock stress [8]. The stress granule discussed in this review refers to the previously mentioned cytoplasmic stress granule. Research on stress granules has brought broad interest, as they not only bridge many cellular processes, including gene transcription and protein translation, but are also related to various human diseases, including cancer, neurodegenerative diseases, and viral infections.

As its name indicates, SGs are not constitutive organelles in the cell under normal growth conditions. SGs are also heterogeneous in composition and dynamics with respect to the cell type and stress conditions. Although research on SGs has spanned over four decades, how SGs are assembled and the physiological and pathological roles they play have only started to be answered in the past few years. Recently, liquid-liquid phase separation (LLPS) has been suggested to be a mechanism of SG formation. It is recognized as a common mechanism for the assembly of many membrane-less organelles. With the identification of hundreds of SG protein components and thousands of RNA substrates, more insight has been revealed into the role of SG-dependent and SG-independent functions of SG-localized biomolecules. More will be learned from SG research in the future.

Research on stress granules dates back to the 1970s when electron-dense structures formed under heat stress in plants and animal cells could be an early indication of stress granule assembly [ 9, 10] . Later research in the 1980s on heat shock proteins (hsp) revealed that hsp70 and many small hsp formed distinct nuclear structures [ 11, 12] , and cytoplasmic heat shock granules were observed in tomato cell cultures and leaves [13]. These structures are resistant to RNase and high salt treatment and are insensitive to cycloheximide treatment. Hsp granules may differ from current knowledge on SGs mainly described in mammalian cell systems. Research on SG has re-emerged recently, and some common features are shared between plant and animal systems. Even in a 1983 report, the authors speculated that “although RNase treatment does not change their protein composition, this does not exclude the possibility that they are ribonucleoprotein particles with a function for storage or protection of mRNAs during heat shock” [13]. Heat shock-induced transcripts that are actively translated are excluded from hsp granules. A study in 1989 characterizing the hsp granule in tomato cell cultures postulated that hsp granules are associated with mRNA and formed from precursor structures and could function as untranslated mRNA storage [14]. We will see in a later section that this model is quite close to the current understanding of SG assembly.

Small hsp aggregates or hsp granules are also observed in vertebrate cells, including hsp24 granules in chicken embryo fibroblast cells [15]. In this study, sodium arsenite (SA) was used to induce hsp24 aggregation, the most applied stressor in the SG field to date. Research on SGs in mammalian cells was re-started when Nancy Kedersha and others linked the RNA-binding protein TIA-1/TIAR with eIF-2α signaling to SG assembly in 1999 [2]. RNA-binding proteins TIA-1 and TIAR have since been regularly used as SG markers in various systems, including mammalian and plant cells [6]. Since then, researchers have observed that not all stress stimuli can cause SG. The duration of stimulation and the concentration of the chemical displayed a dose- and time-dependent effect on SG induction, indicating a threshold of SG formation inside cells. PolyA RNA and RNA-binding proteins are enriched in SGs, but not all RNA-binding proteins can localize to SGs, indicating the specificity of SG composition. SGs formed under different stress stimulations may contain different components. For example, HSP27 exists only in heat shock-induced SGs but not in sodium arsenite-induced SGs [6].

SGs are not static structures, and early studies recognized the dynamic nature of SG components and suggested that SGs may function as a triage for mRNA fate for translation, decay, or recycling [ 7, 16– 18] . SGs disassemble during mitosis, possibly due to CDK- or DYRK3-mediated phosphorylation of key SG proteins [ 19– 21] . Since 2000, more SG proteins have been identified, including HuR [22], FMR1 [23], G3BP1 [24], Ago2 [25], and many others, among which most are RNA-binding proteins [26]. Their connections to cell survival [2], apoptosis [27], viral infection [28], and cell signaling [29] have been suggested. SGs are physically and functionally connected to another cellular ribonucleoprotein (RNP) granule called the p-body [ 30, 31] . p-Bodies contain diverse RNA decay and translation silencing machinery [32]. Many protein and RNA components of SGs and p-bodies are shared. It has been suggested that the difference in protein-protein, protein-RNA, and RNA-RNA interaction networking is the underlying determinant of SG and p-body identity [33]. The formation of SGs is closely linked to cellular RNA metabolism and has substantial implications for human disease pathogenesis. Unlike the nucleolus and ribosomes, SGs are not constitutive organelles in cells. The way they assemble in response to stress remained a mystery until the recent re-emergence of the concept of liquid-liquid phase separation, which shed light on the assembly mechanism of these membrane-less organelles (MLOs) in cells [34].

A genome-wide RNAi screening in 2008 for SG and p-body regulators in mammalian cell lines revealed an essential role of GlcNAC modification of ribosomal proteins in these RNP granule dynamics [35]. In 2016, Roy Parker’s group [36] reported the first purification of SG core structures from yeast and mammalian cells and provided a comprehensive database of SG proteomes. This study expanded the SG proteome to more than 400 proteins. The SG proteome is enriched with RNA-binding proteins and shows the enrichment of proteins with disordered regions. A later RNA-sequencing study revealed that most RNA inside SGs is under-translated mRNA [37]. The length of mRNA correlates well with SG localization. In 2020, three papers revealed that the liquid-liquid phase separation between G3BP protein and ribosome-free mRNA is the underlying mechanism of SG assembly and provided a network framework for MLO studies [ 33, 38, 39] . The yeast model for SGs and p-bodies has revealed many fundamental principles of RNP granules [26]. Common stress stimulations include sodium azide and glucose deprivation. The granule assembly, kinetics, and compositions vary in a stress-dependent manner, consistent with what is observed in mammalian and plant systems.

What is the physiological relevance of SGs if they can only be induced by harsh stresses, such as 0.5 mM sodium arsenite or 30 min of 43°C heat shock? These early robust stimuli-induced SG models revealed some common biomolecule interactions and assembly mechanisms of SG and related RNP granules. Nevertheless, the formation and function of SGs will be better understood under specific physiological or pathological conditions. There are a couple of examples showing the relevance of SG to physiology and pathology. First, SGs have been observed in ischemic brain regions in a mouse model, indicating that SGs can be formed in an *in vivo* context [40]. Second, SG formation is commonly observed in tumor tissues, probably due to the low oxygen and nutrient-deprived stress microenvironment [ 41, 42] . Elevated SG formation may contribute to the survival advantage in U2AF1 mutation-dependent myeloid malignancies [43]. Clinically, trioxide arsenite has been successfully used for acute promyelocytic leukemia (PML) treatment [44]. Third, virus infection, especially RNA virus infection, can induce SG assembly. Viral infection-induced SGs exhibit distinct dynamics with respect to the viral strategies evolved to target host SGs, which will be further elaborated on in later sections. Fourth, plants cannot move. SGs could form upon elevated temperatures during drought and climate change. Whether the adaptation of SG formation will be a strategy for crop engineering may be further explored in the future.

### The re-emerging concept of liquid-liquid phase separation (LLPS) in biology

Liquid-liquid phase separation (LLPS) is a form of biomolecular phase separation, as illustrated effectively in the landmark paper published by Clifford Brangwynne, Anthony Hyman, and others in 2009 [45]. The study ruled out active transport and passive degradation as mechanisms of P granule asymmetric localization during *C*. *elegans* early embryo development, proposing a dissolution-condensation process of P granule components as the underlying mechanism of the asymmetric P granule distribution. Since then, the field has expanded with numerous examples of LLPS features within and outside cells [ 46, 47] . The term “biomolecular condensate” was initially proposed by Michael Rosen, Anthony Hyman, and others, referring to any structures enriched with biomolecules without lipid membrane confinement [34]. Just as water molecules exist in three common states (gas, liquid, solid) in the Earth′s natural environment, cellular biomolecules can also form condensates with varying material properties [47]. These properties can change over time for a given condensate, exerting different functions. Researchers often describe the material property as “liquid-like” or “solid-like”, but there is no consensus threshold of “liquidness” to distinguish the liquid state from the solid state.

While the biomolecular condensate field is new and rapidly expanding, the concept has its roots in biological research dating back to 1899 when E. B Wilson [48] suggested that the “vacuole” in cells is “liquid-like” due to its ability to fuse and exchange materials. These membrane-less organelles could serve as crucial compartmentalization mechanisms for cell organization. Biomolecule phase separation, especially protein phase separation, has been a common observation in laboratories, particularly in structural biology labs, during the process of obtaining crystals for X-ray crystallography studies. For instance, lysozyme solution has been observed to phase separate into droplets as the temperature decreases [49]. This effect can be enhanced by adding a crowding reagent, such as PEG or Ficoll. The phase separation of eye lens crystallin in the presence of crowding reagent and at reduced temperature was documented in the 1970s [ 50– 54] . Crystallin is abundant in the eye lens, and aberrant phase transitions caused by crystallin can lead to cataracts. LLPS of hemoglobin may underlie another protein condensation disease, sickle cell anemia [55]. It is also suggested that the dense liquid droplet interfaces and aggregate interfaces may play an important role in crystal nucleation. LLPS, crystallization, gels, and aggregate formation are commonly observed in aqueous solutions when investigating salting-out effects with ammonium sulfate and sodium chloride on substances including ovalbumin, ribonuclease A, soybean trypsin inhibitor, and lysozyme [56].

P granules, or polar granules, are germ-line-specific structures crucial for germ cell fate determination in various organisms. The partitioning of P granules from a homogenous state in the fertilized embryo to germ cell localization after the first cell division remained mysterious until the dissolution-condensation model was proposed in 2009 [45]. This process was successfully reconstituted in the test tube in 2016, showing that the MEX-5 RNA gradient regulates PGL3-RNA condensate through an RNA competition mechanism [57]. Later work from Geraldine Seydoux′s group showed that the LLPS of intrinsically disordered MEG3 proteins helps retain RNA in the gel-like sub-compartment of P granules, suggesting a heterogenous state of P granules [ 58– 60] . P granule components are eventually cleared by autophagy during *C*. *elegans* embryogenesis [61]. The material properties of condensate can be regulated by post-translational modifications. For example, mTOR regulates the material properties of PGL granules to modulate their autophagic degradation [62]. Thus, LLPS is not only involved in the biogenesis of P granules and their subcellular localization but also plays important roles in their function and clearance.

Intrinsically disordered protein (IDP) or intrinsically disordered region (IDR)-containing proteins are widely identified as initiators of LLPS. The features encoded in the IDR sequence ultimately determine the LLPS capacity. Fused-in-sarcoma (FUS) is well studied and has been used to reveal that positively charged arginine and aromatic residues are critical for FUS homotypic LLPS [63]. These residues are suggested to act as stickers separated by spacers, and their distribution is important for LLPS [ 64, 65] . The valence encoded in the IDRs is critical for the critical threshold of phase separation. The modular domain is another module that can facilitate the phase separation of biomolecules. Proteins with multiple binding domains and nucleic acids, such as mRNA, exhibit a strong capacity for phase separation. Multivalent signaling proteins with SH3 and PRM domains are used as examples to demonstrate the importance of valency in modular protein phase separation [66].

RNA-binding proteins with IDR regions serve as nucleators for multiple condensates, and mutations in several such proteins cause amyotrophic lateral sclerosis (ALS). Investigations into the pathogenic mechanisms of these ALS mutations have spearheaded our understanding of the functions and molecular framework of biomolecular condensation. Well-studied examples include hnRNPA1, FUS, and TDP43 proteins [ 67, 68] . Since the initial recognition of phase separation regulation in ALS, the concept has been introduced and proven in many other neurodegenerative diseases [ 69, 70] . Studies of P granules under both developmental and physiological conditions, as well as investigations into aberrant phase separation in pathologic conditions, provide platforms and guidelines for all future investigations.

The re-emergence of the LLPS concept in biology has been noted in various biological processes, including cell signaling, chromatin organization, gene transcription, splicing, nuclear pore transport, and membrane invagination and association. The field of biomolecular condensates is multidisciplinary and has benefitted from biophysical studies, polymer biochemistry, computational simulations, and other disciplines. For any identified condensate, several aspects need to be examined: what are the cellular conditions or biological context it formed? What are the key components in the condensate, and what is the biochemical/biophysical basis for condensate formation? Are there a few scaffold proteins or RNAs for condensation? How are condensation processes regulated, and could they be harnessed for therapeutic or bioengineering purposes? We refer readers to the many recent reviews published on LLPS and MLO studies [ 34, 71– 74] , as well as many excellent works on viral-host interactions [ 75– 80] . We will illustrate these points using the example of stress granule research, showing how the knowledge has been gained and used for targeting viral infections and the potential directions for future research.

### Signaling of SG formation

Stress granules can form in response to various stimuli, and the general shutdown of protein translation is critical for SG formation. The translation initiation factor eIF2α occupies a key node of this signaling pathway, converging upstream signals with four kinase branches, including general control nonderepressible 2 (GCN2), protein kinase RNA-like ER kinase (PERK), heme-regulated inhibitor (HRI), and protein kinase R (PKR). Activation of any of these kinases leads to the phosphorylation of eIF2α and reduced cap-dependent protein translation [81]. PKR, which contains a dsRNA-binding domain, is activated upon dsRNA binding. HRI is a heme sensor that can respond to reactive oxygen species (ROS). PERK is activated by the accumulation of misfolded proteins in the ER and during hypoxia. GCN2 is activated by amino acid deprivation and UV irritation. There is also an eIF2α-independent pathway through the inhibition of the eIF4A helicase [ 82, 83] . These studies showed that translation inhibition, which does not induce eIF2α phosphorylation, can also lead to SG formation.

**Figure 1:**
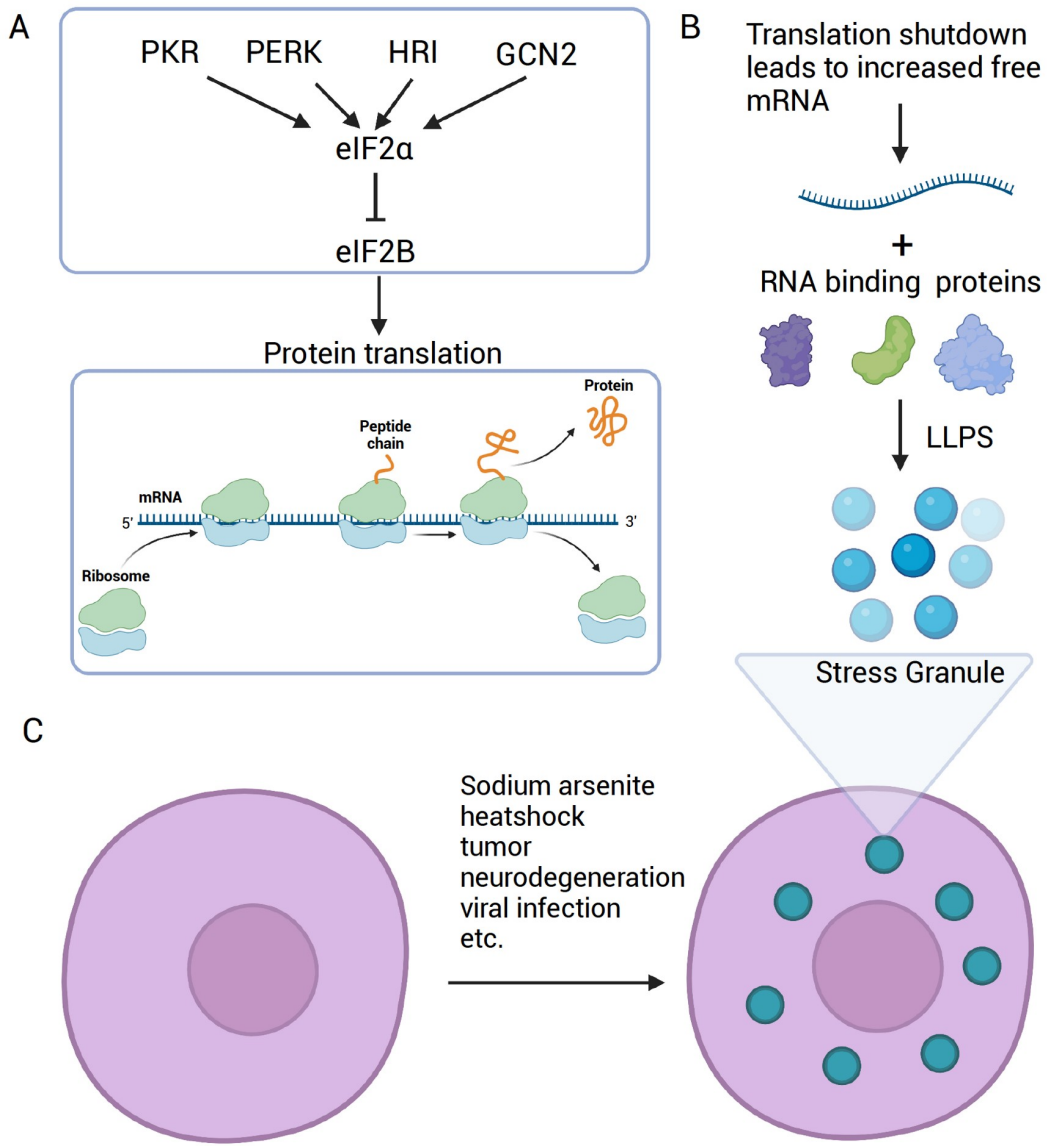
Figure 1 Signaling in SG assembly (A) Four upstream kinases for eIF2α and their effect on the protein translation process. (B) The increased mRNA level in the cytoplasm due to translation shutdown coupled with various RNA-binding proteins can undergo liquid-liquid phase separation to form SGs. (C) Various environmental and internal stresses can induce SG formation in cells.

Since the early indication in 1989 that SGs assembled from precursor structures, further identification of protein and RNA composition and the successful reconstitution of SG in test tubes have provided evidence for the current model of SG assembly. Generally, following the inhibition of translation initiation and ribosome run-off from mRNA, the pools of untranslated mRNA bound by various RNA-binding proteins increase. These proteins include G3BP1/2, Caprin1, UBAP2L, DDX3, small subunits of ribosomes, and many other abundant RNA-binding proteins in the cytoplasm. During stress, nuclear-cytoplasmic transport is perturbed, leading to the accumulation of many nuclear RNA-binding proteins in the cytoplasm, including TIA1/TIAR, FUS, and TDP43. The increased mRNA-RNA binding protein (RBP) interaction, RNA-RNA interaction, and protein-protein interaction lead to the phase separation of these RNP complexes, forming the nucleation cores of SG cores ( Figure 1).

The initial observation of SG formation under stress correlates with a general shutdown of translation. Translation inhibitors, including emetine and cycloheximide, either block SG formation or promote SG disassembly. Conversely, puromycin, which promotes ribosome disassembly, enhances SG assembly. Thus, the shutdown of translation and ribosome run-off are indispensable for SG formation [16]. Single molecular imaging has revealed the dynamic exchange of mRNA between SGs and p-bodies, along with changes in mRNA conformation inside SGs [ 84, 85] . There is also evidence to suggest that mRNA can be translated within SGs [84]. However, further investigation is required regarding the translation activity of mRNA inside condensates at the organismal level.

Sodium arsenite (SA) is the most commonly used stimulant for inducing SG formation in the field thus far. SA can cause various downstream effects, including ROS generation and eventually eIF2α phosphorylation. This effect is primarily mediated through the heme-regulated inhibitor (HRI) kinase [86]. Upon viral infection, dsRNA intermediates can activate several host dsRNA sensors, including RIG-1 and TLR3. These sensors activate the IRF3/7-dependent interferon response as well as the PKR kinase and RNase L pathway, leading to eIF2α phosphorylation and host translation shutoff. Antiviral mRNA escapes from this translation at the early stage of viral infection. Many interferon-related innate immune response genes are upregulated, thereby escaping degradation by RNase L. RNase L can mediate rapid mRNA turnover even before eIF2α phosphorylation occurs. Noncanonical SGs are formed during viral infection with smaller G3BP granules and PABPC1 nuclear translocation. G3BP forms an RNase L-dependent body (RLB) distinct from canonical SGs [87]. RNase L is partially responsible for eIF2α phosphorylation, as the level of phosphorylated eIF2α is reduced in RNase L KO cells. PKR is only partially responsible for poly(I:C)-induced eIF2α phosphorylation, as phosphorylated eIF2α is observed in *PKR*-KO cells, albeit at lower levels than in wild-type cells. These results indicate that an additional kinase downstream of RNase L is involved [87].

The relationship between canonical SG proteins and viral infection remains unclear. PKR is activated in response to dsRNA recognition, and early evidence showed the localization of PKR in SGs. Two recent reports revealed that activated PKR forms novel cytoplasmic clusters distinct from SGs and proposed roles for these clusters in signaling activation [ 88, 89] . In response to dsRNA stimulation, such as viral infection, PKR can form a novel cytoplasmic condensate called dsRNA-induced foci (dRIFs). Multiple dsRNA-binding proteins, including ADAR1, Stau1, NLRP1, and PACT, localize to dRIFs [89]. The precise mechanism of PKR assembly and function, however, remains to be determined. It is hypothesized that the PKR condensate may facilitate the detection of low amounts of dsRNA and activation of PKR, which is consistent with early reports that SGs regulate PKR activation [90]. Another report indicated that PKR forms clusters in response to dsRNA, but disruption of the PKR cluster could further enhance eIF2α phosphorylation. This suggests that the PKR cluster may buffer the activation of PKR activity [88]. Other dsRNA sensors, including RIG-I, MAD-5, and OAS, have also been reported to localize to SGs for their activation. Further investigation is needed to understand the exact mechanism and function of these structures.

In the previous section, we examined the upstream signaling pathways leading to SG assembly established in the past few decades. This can form the basis for later perturbation of SG formation during viral infection and other human pathologies. In the following sections, we will discuss the critical roles of proteins and RNA in SG assembly and dynamics.

### Role of RNA-binding proteins in SG formation and regulation

Several proteins have been reported as essential for SG assembly over the past few years, including TIA1/TIAR, G3BP1/2, UBAP2L, PRRC2C, and CSDE1. We propose that these proteins function together in a protein-RNA network, with different contribution from each protein in the network. We aim to reconcile the contradictions in the literature using the network theory. A quantitative analysis of yeast p-bodies revealed seven abundant proteins inside the p-body, correlated with a high partition coefficient (PC)>30 [91]. More than 400 proteins have been identified in human SGs, and the abundant SG proteins likely play more critical roles in SG assembly and regulation [92]. RNA sequencing of the G3BP SG core identified only 185 genes with more than 50% of RNA molecules inside SGs, whereas almost all mRNA could partition into SGs to varying extents [37]. Since there are no experimental data on the partition coefficient for SG proteins, we curated a list of core SG proteins and estimated their total cellular concentrations based on published data. The databases used are for HEK-293 and HeLa cells [ 93, 94] . We further added paralog proteins to the list. The partition coefficient will determine the enrichment of each protein inside the SG. Nevertheless, this list can provide a glimpse into the role of each SG protein in assembly, as described in the following section ( Table 1). G3BP1/2, Caprin1, DDX3, UBAP2L, and CSDE1 are abundant proteins, consistent with their reported key roles in SG assembly. We suggest that these proteins could serve as the core proteins for *in vitro* SG reconstitution. Investigation into the features of these proteins could provide principles for SG assembly and could further guide the study of SG assembly and dynamics.

**Table:** **Table 1** Cellular protein concentration of the core SG network proteins*

SG protein	HEK-293 (μM)	Hela (μM)	Description
HNRNPA2B1	20.336	8.347	Heterogeneous nuclear ribonucleoproteins (hnRNPs)
SRSF3	7.080	2.442	Splicing factor
KPNB1	4.510	2.227	Karyopherin Subunit Beta 1
EIF3I	3.921	0.854	Eukaryotic Translation Initiation Factor 3 Subunit I
PPP2R1A	3.388	0.672	Protein Phosphatase 2 Scaffold Subunit Aalpha
G3BP1	3.062	0.624	RNA binding protein
DDX3X	2.879	1.129	DEAD-Box Helicase 3 X-Linked
EIF3G	2.577	1.152	Eukaryotic Translation Initiation Factor 3 Subunit G
RAB1A	2.523	2.965	Ras superfamily of GTPases
CAPRIN1	2.332	0.352	RNA binding protein, direct interactor with G3BP1
EIF3D	2.032	1.034	Eukaryotic Translation Initiation Factor 3 Subunit D
G3BP2	1.934	0.822	RNA binding protein
EIF3E	1.668	0.316	Eukaryotic Translation Initiation Factor 3 Subunit E
CSDE1	1.622	0.317	Cold Shock Domain Containing E1
UBAP2L	1.415	0.655	Ubiquitin Associated Protein 2 Like
CBX1	1.362	0.383	Chromobox Protein Homolog 1
TIAL1	1.146	0.137	TIA1 Cytotoxic Granule Associated RNA Binding Protein Like 1
DDX19A	0.957	0.436	DEAD-Box Helicase 19A
FXR1	0.840	0.598	FMR1 Autosomal Homolog 1
UPF1	0.746	0.294	RNA Helicase And ATPase
ATXN2L	0.719	0.174	Ataxin 2 Like
NXF1	0.646	0.153	Nuclear RNA Export Factor 1
TIA1	0.631	0.057	TIA1 Cytotoxic Granule Associated RNA Binding Protein
PRRC2C	0.522	0.078	Proline Rich Coiled-Coil 2C
TAF15	0.508	0.051	TATA-Box Binding Protein Associated Factor 15
NUFIP2	0.486	0.179	Nuclear FMR1 Interacting Protein 2
TRIM25	0.473	0.533	Tripartite Motif Containing 25
USP10	0.473	0.095	Ubiquitin Specific Peptidase 10
UBAP2	0.413	0.101	Ubiquitin Associated Protein 2
FMR1	0.403	0.055	Fragile X Messenger Ribonucleoprotein 1
POLR2B	0.394	0.097	RNA Polymerase II Subunit B
NUP98	0.360	0.271	Nuclear Pore Complex Protein
FXR2	0.311	0.091	FMR1 Autosomal Homolog 2
ATXN2	0.271	0.055	Spinocerebellar Ataxia Type 2 Protein
PRRC2A	0.178	0.028	Proline Rich Coiled-Coil 2A
PPP1R10	0.133	0.032	Protein Phosphatase 1 Regulatory Subunit 10
PRRC2B	0.096	0.013	Proline Rich Coiled-Coil 2B
MACF1	0.069	0.021	Microtubule Actin Crosslinking Factor
HDAC6	0.061	0.013	Histone Deacetylase 6
TRIM56	0.013	0.039	E3 Ubiquitin-Protein Ligase TRIM56

*Note: The SG core network proteins comprising 36 proteins and related paralogs are included in this Table [38]. The protein concentration was estimated in HEK-293 cells [94] and HeLa cells [93].

#### TIA1/TIAR

TIA1 (T cell restricted intracellular antigen) is an RNA-binding protein involved in mRNA splicing and translation and is typically localized in the cell nucleus. TIA1 contains three N-terminal RRM domains and a C-terminal disordered low complexity domain/prion-like domain. TIA1/TIAR can translocate to the cytoplasm to form SGs upon various stimulations and have been widely used as markers for SG labeling [2]. Early reports showed the importance of TIA1 in SA-induced SG formation, and the overexpression of the TIA1 low complexity domain (LCD) can impair SG assembly [ 16, 95] . Due to the redundancy between TIA1 and TIAR, attempts to create double knockout (dKO) cells failed to produce viable clones in HEK-293 cells. However, with an inducible dKO strategy, TIA1/TIAR dKO cells were obtained, which showed mis-splicing of mRNA, cell cycle arrest, activation of EIF2AK2-mediated stress response, and apoptosis [96]. Interestingly, dKO led to spontaneous SG formation, suggesting that TIA1/TIAR may not be essential for SG formation.

TIA1/TIAR has been shown to bind to West Nile and Dengue virus RNA, facilitate viral genome replication, and inhibit SG formation, preventing host protein translation shutoff [97]. During polio infection, SGs are formed with many canonical SG markers, translation initiation factors, RNA-binding proteins, and polyA mRNA. As the infection progresses, the composition of SGs is remodeled with the disappearance of translation initiation factors and polyA RNA. TIA1 was reported to be one of the residual proteins left in the later stage of remodeled SG during polio infection [98]. Similar remodeled SGs were observed in vesicular stomatitis virus (VSV)-infected cells, and TIA1 and PCBP2 are components of these noncanonical SGs [99].

TIA1 contains a prion-like domain (PrLD), an intrinsically disordered domain. TIA1 protein can undergo LLPS *in vitro* and shows dynamic exchange with the surrounding environment. TIA1 mutations can lead to ALS, and point mutations within the PrLD can accelerate the liquid-to-solid transition of the condensate [100]. These same TIA1 mutations can slow the recovery of SGs after stress removal and promote the accumulation of non-dynamic SGs. TIA1 LLPS facilitates RNA-triggered TAU LLPS in cells and promotes TAU oligomer formation, suggesting that RNA metabolism is a prevalent mechanism for various neurodegenerative diseases [101]. Zinc ions bind to the RRM domain and promote not only protein oligomerization but also LLPS of the TIA1 and TAU proteins [ 102– 104] . Changes in zinc concentration and localization occur under stress and viral infection. These studies highlight the critical role of metal ions in RBP protein LLPS and suggest potential intervention pathways.

#### G3BP

G3BP1 has been used as a bona fide marker for SG for decades. G3BP1 mainly localizes in the cytoplasm and is abundant in many cell types. G3BP1/2 is also broadly expressed in various tissues. The combined concentration of G3BP1/2 can reach over ~1.8 μM in U2OS cells, ~2.2 μM in Hela cells [33], and ~5 μM in HEK-293 cells [94]. Human *G3BP1* and *G3BP2* encode proteins of 466 and 488 amino acids, respectively, and are highly paralogous. Human *G3BP2* encodes a shorter isoform with 449 amino acids. G3BP1/2 is redundant in SG formation and has been used as a bait protein for SG core isolation and transcriptome analysis. G3BP1/2 has an N-terminal NTF2L domain, indicating potential involvement in nuclear-cytoplasmic transport. G3BP can interact with various NUP proteins, and NUP can be recruited into SGs under stress, which has implications for nuclear-cytoplasmic transport [105]. G3BP1/2 has C-terminal RNA-binding motifs consisting of the RRM and disordered RGG domains. The middle half of the G3BP protein is disordered, with an acidic IDR1 and a positively charged spacer IDR2 between IDR1 and RBD.

Combining the genome-wide RNAi screening in cell line and the SG proteome identified by the Parker group, we identified a protein network underlying SA-induced SG formation in the U2OS cell line [38]. After analysis, an additional 36 protein core networks containing G3BP1 emerged. This network of proteins has denser connections than the SG proteome. A knockout (KO) screening in cells showed that *G3BP1*/ *2*-dKO cells exhibited complete blockage of SG formation upon sodium arsenite treatment. This study indicated that G3BP1/2 might function as a scaffold for SG formation under SA treatment. Other SG proteins were tested to function as clients recruited to SGs, but they played a less critical role in SG assembly. This scaffold-client model has been examined in many cellular MLOs [106]. For example, NEAT1 lncRNA is essential for the formation of nuclear paraspeckles [107], PML protein is essential for PML body formation, and SPD5 is essential for *C*. *elegans* centrosome assembly [ 108, 109] .

G3BP1 protein can undergo LLPS *in vitro* with a crowding reagent or with RNA. The features underlying crowding and RNA-triggered phase separation are different. We believe that the G3BP-RNA LLPS system can better recapitulate SG in cells. Multivalency encoded in the G3BP protein and RNA determines the phase separation threshold and SG formation in cells. The interaction valence is critical in many LLPS systems. The multivalency is encoded in the tandem RNA-binding motifs in G3BP1, as well as the length of RNA. Both RNA-binding motifs are required for LLPS, and the valence of arginine in the RGG domain is also important. Mutation of 5 out of 11 arginine residues in G3BP1 abolishes RNA-triggered LLPS, but crowding-induced LLPS can still occur. Longer RNA can better facilitate G3BP1 LLPS. Single-strandedness is a crucial feature, but G3BP shows less sequence specificity for the RNA sequence [ 38, 39] .

The valence, but not the binding affinity, matters. With the SWAP of RBD with the KH motif to create chimeric G3BP proteins, the rescue of binding affinity cannot rescue SG formation, but a valence of no less than two motifs is required for G3BP LLPS. Network theory can also help explain the contribution of other SG proteins to SG formation. Caprin1 and TIA1 are binding partners of G3BP, and both can promote G3BP1-RNA LLPS, indicating positive cooperativity. It has long been recognized that USP10 is a negative regulator of SG, with inhibition of SG by USP10 overexpression [ 33, 38] . This study showed that the collected protein-protein and protein-RNA interactions are essential for SG formation. Some nodes could increase the valence of the system, and nodes that decrease the valence are thus negative regulators of the network. This work provided a framework for investigating the assembly and regulation of MLOs. Studies in the past few years have shown that many viral factors, such as SARS-CoV-2 nuclear capsid protein N, can interact with G3BP1 and perturb the SG assembly of host cells [110].

Multiple pieces of evidence have shown the importance of post-translational modification (PTM) of G3BP in regulating SG dynamics. Upon SA treatment, G3BP undergoes arginine-demethylation within their RGG RNA-binding domains, likely increasing their RNA binding and promoting SG assembly [111]. G3BP1 can be phosphorylated at serine 149 in the acidic IDR1 region, further facilitating the close conformation of the G3BP1 molecule in the inactive state. The phosphorylation level decreases upon SA treatment, with an increase of free mRNA in the cytosol to drive LLPS with mRNA [ 24, 38] . G3BP plays a central role in SG biology, and multiple ALS-related genes and mutations are related to SG dynamics. A recent finding revealed that mutations in G3BP1/2 can lead to neurodevelopmental disorders [112]. These mutations disrupt network formation and SG formation, further implicating the importance of RNA metabolism in neurologic diseases. G3BP1 was also reported to function in dsRNA-mediated RNA decay [113], ribosome recycling [114], mTOR regulation [115], and the innate immune response [ 116, 117] . Many of these functions are suggested to be independent of the SG assembly capacity of G3BP1.

#### UBAP2L, PRRC2C, and CSDE1

UBAP2 and UBAP2L are SG proteins. They are homologous proteins in the human proteome containing ubiquitin-binding and RNA-binding domains. UBAP2L is considered one of the key proteins required for SG formation, and *UBAP2L* KO alone significantly reduced SG formation in U2OS cells. Proximity labeling with BioID and APEX2 fused to G3BP1 identified UABP2L/UBAP2 as SG components [ 118, 119] . A KO cell analysis of more than 20 SG genes showed that G3BP1/2 could completely block SG formation upon SA treatment [38], whereas UBAP2L, PRRC2C, and CSDE1 are three other genes exhibiting defects in SG formation [ 118, 120] . Quantitative analysis showed significantly decreased SG formation in the three KO cell lines, indicating that UBAP2L, PRRC2C, and CSDE1 are important nodes in the SG-forming protein networks [ 118, 121] . UBAP2L contains UBA and RGG domains required for optimal SG assembly. The FG motif in the middle of UBAP2L is suggested to interact with G3BP, and deficiency in G3BP interaction also leads to reduced SG formation, suggesting cooperativity between UBAP2L and G3BP in SG assembly.

UBAP2L can still form granules in *G3BP1*/ *2* dKO cells [121], which are positive for p-body markers. The interaction between UBAP2L and G3BP and their roles in SG formation need further investigation. G3BP has two homologs in vertebrate species, and only one homolog can be identified from the amino acid alignment in many invertebrate species. The UBAP2L homolog PQN-59 and G3BP homolog GTBP1 in *C*. *elegans* have been shown to localize to SGs under heat shock stress in the germline and embryo stages. Genetic deletion mutants showed the dispensability of PQN-59 and GTBP1 in SG formation in the worm model [122]. The G3BP1 homolog in Drosophila, RIN, can also localize to SGs upon SA treatment in the intestinal progenitor cell system. Genetic ablation of RIN shows the non-essentiality of RIN for SG formation in this model, suggesting the context dependency of this protein in SG formation [123].

#### Other proteins in SG

Many SG proteins are nuclear proteins. The nucleolus has a tight connection with SG formation; many nucleolus proteins shuttle to SGs in the presence of stress and are regulated by several cell signaling pathways [ 124, 125] . Nuclear-cytoplasmic transport defects in the SG proteins TDP43, FUS, hnRNPA1, and several other RNA-binding proteins can lead to the pathogenesis of ALS. The disruption of RNA metabolism upon stress is connected to nuclear and cytoplasmic activities. Many cancer cell lines have an enlarged nucleolus to confer higher protein translation activity, which correlates with increased SG assembly in many cancer cell lines.

### Role of RNA in SG formation

RNP granules are prevalent under physiological and pathological conditions [ 126, 127] and can even be secreted from cells. For example, the RNA-binding protein MRJP-3 forms RNA granules in honeybee royal jelly [128], and YB1 condensate regulates selective miRNA sorting into exosomes [129]. RNA functions as a scaffold and is required to assemble and maintain multiple RNP granules, including SGs, Cajal bodies, nucleoli, and nuclear speckles [130]. Degradation of cellular RNA with RNase L activation leads to the disassembly of these RNP granules. The requirement of RNA in condensation not only reflects its scaffolding role in RNA-protein interactions. RNA-RNA interactions are also essential for SG formation, and the exact mechanism likely applies to other RNP condensates [131]. RNA can undergo LLPS itself, and the G4C2 repeat-containing transcript from the *c9orf72* gene can phase separate *in vitro*, form RNA foci in patient neurons, and exert toxicity to cells. The exact sequence and repeat number are critical for repeat RNA phase separation [132]. RNA homopolymers and total cellular RNA can also form assemblies in test tubes. mRNA enriched in the *in vitro* RNA condensate can recapitulate the RNA transcriptome of SG in cells [133]. RNA helicases are ATP-dependent RNA-binding proteins that disrupt RNA-RNA interactions. eIF4A and DDX19A have been shown to inhibit SG formation [134]. Collectively, these studies demonstrate the intrinsic condensation capability of RNA molecules.

Super-resolution microscope imaging reveals the heterogeneity of RNA clusters inside SG and germ granules [ 36, 135] . The PGL3 liquid phase demixes from the MEG3 gel-like phase in the P granule, and RNA is enriched in the gel-like phase [ 59, 136] . Enrichment into the RNP granule is sequence-dependent. RNA inside germ granules can form homotypic clusters independent of sequence [137]. RNA does show specificity in triggering protein LLPS. Cytoplasmic Whi3 condensates of the multinucleate filamentous fungus *A*. *gossypii* form two types of condensates with *BIN1* and *CLN3* mRNA. The secondary structure of RNA determines the specificity of the condensate [ 138, 139] . The secondary structure of SARS-CoV-2 genomic RNA also determines condensation with the nucleocapsid N protein [140]. RNA modification also plays a role in SG formation. m6A modification in mRNA can regulate the stability and translation activity of target mRNA. YTHDF family proteins can bind to m6A-modified mRNA and localize to SGs. LLPS between the YTHDF protein and m6A-modified RNA contributes to SG assembly [141]. However, a recent report showed a limited role of m6A modification in mRNA partitioning into SGs [142].

What is the concentration of free RNA in cells? It is estimated that the concentration of cytosolic RNA in mammalian cells can reach 80-300 ng/μL under stress [131]. One mechanism for viral replication is competition with host RNA for the translation machinery. Single-molecule imaging has revealed distinct phases of RNA recruitment in SGs, characterized by initial dynamic contact and eventual stable incorporation. The dynamics of RNA within SGs are generally slower than those of SG proteins. SGs and many other RNP granules collectively assemble from the summation of multivalent interactions between RNPs [143]. In addition to mRNA, which is a prominent RNA species in SG assembly, noncoding RNA is also detected in SG [37]. For instance, NORAD is one of the most enriched lncRNAs in SG, and it has been shown to partition into SG.

### Regulation and function of SGs

LLPS provides a solid framework for studying the dynamics of SGs, whose disassembly plays critical roles in various pathological conditions. Aberrant clearance of SGs can lead to the formation of fibril-like protein aggregates, resulting in loss of function and toxicity. SG disassembly occurs mainly through two pathways: degradation via the autophagy/UPS pathway and recycling of SG components to a diffused state. ALS mutations often result in the stabilization of SG and delayed clearance. Similarly, SG dynamics are regulated during viral infection, with active remodeling of SG composition by targeting the phosphorylation state of eIF2α or cleavage/hijacking key SG proteins.

Cytoplasmic SG localization can regulate a protein’s function by sequestering the protein away from normal localization. For example, 14 out of 20 tested NUPs, which form nuclear pore complexes, have been shown to localize to SGs. Nuclear-cytoplasmic transport is defective in cells under stress when SG formation occurs [105]. Inhibition of SG formation can suppress the toxic effect of dipeptide repeats, implicating the role of SG in C9-ALS/FTD. Nuclear-cytoplasmic transport can be perturbed by artificial beta-sheet protein aggregates in cells, even though the relationship between these aggregates and SG is unclear [144]. Cytoplasmic aggregates, not nuclear aggregates, enrich the IDR-containing import and export machinery, impairing nuclear-cytoplasmic transport. The toxicity is lost if the aggregated proteins are targeted to the cell nucleus, suggesting that the localization of the protein aggregates is critical. Nuclear-cytoplasmic transport defects have been implicated in many neurodegenerative diseases, suggesting a common underlying mechanism.

The composition of SG varies under different stresses and in different cell lines. An important and closely related analog of SG is the neuronal granule in the nervous system. Neuronal granules are constitutively present in neurons, primarily in neurites, and contain many RNA-binding proteins commonly identified in arsenite or heat shock-induced SGs, including FMR1 [145] and G3BP1 [ 146, 147] .

SGs are also connected to the ER network, and the membrane structure plays a crucial role in SG division [148]. ER contact sites are the physical division point of SG and p-bodies. The surface tension determines the low chance of spontaneous fission of the MLOs [149], and the membrane contact can facilitate this process. This highlights the critical connection between MLOs and membrane structures. Another example of an MLO-membrane relationship is the hitchhiking of SG on endosomes/lysosomes for trafficking in neuron cells [150]. ANXA11 contains an IDR and a lipid-binding region that bridges MLO with endosomes/lysosomes. ALS-related mutations disrupt SG localization and reduce mRNA transport and local translation in neurons [ 151, 152] . Whi3 RNA condensate is also associated with the ER in *A*. *gossypii*, suggesting that the membrane contact site can regulate the size and dynamics of MLOs [153].

## Viral Protein Granules-Phase Separation in Viral Infection

**Figure 2:**
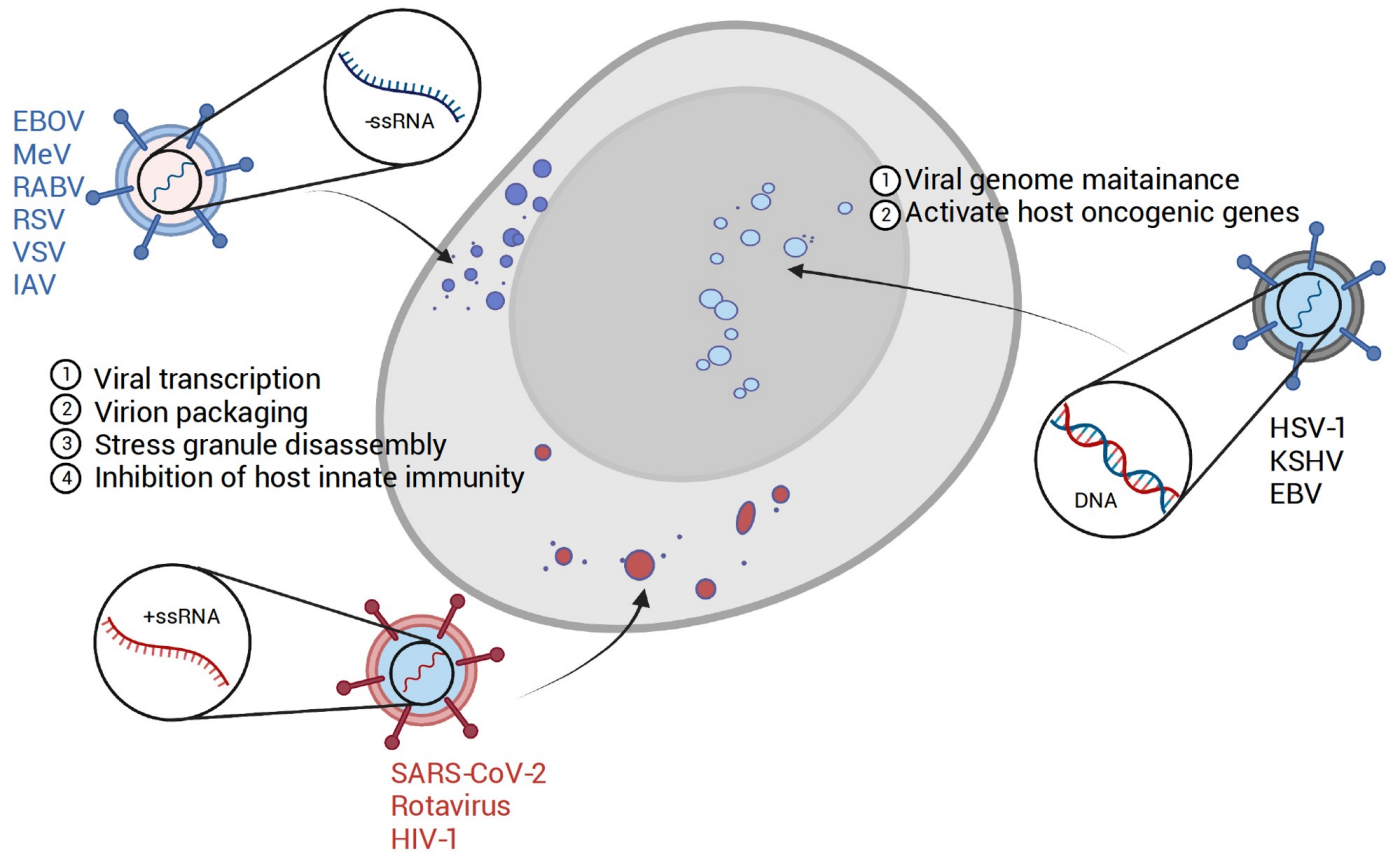
Figure 2 Various viral condensates formed inside cells Positive-strand RNA viruses, such as SARS-CoV-2 and Rotavirus; negative-strand RNA viruses, such as Ebola virus, measles virus, rabies virus, and RSV; and DNA viruses, such as HSV1, KSHV, and EBV, encode proteins able to form condensates in cells during the viral life cycle.

Viral infection significantly remodels the host’s intracellular signaling network and structures. The formation of specialized structures enriched in viral/host protein/nucleic acids, also known as inclusion bodies (IBs), viral replication compartments (vRCs), viral factories, virosomes, or viroplasms, has been extensively investigated. With the rapid growth of understanding in LLPS, these structures are now increasingly recognized as membrane-less biomolecular condensates. In this section, we review recent advances in the role of liquid-liquid phase separation in viral replication and virion assembly, highlighting phase separation as an essential and common strategy supporting viral replication ( Figure 2).

### Negative-stranded RNA viruses

*Mononegavirales*, established in 1991, includes viruses with non-segmented, linear, single-stranded, negative-sense RNA genomes. According to the International Committee on Taxonomy of Viruses (ICTV), 11 families are classified [154]. These large genera include several highly pathogenic viral species, such as Ebola virus (EBOV), Measles virus (MeV), and Rabies virus (RABV), which have been reported to complete their replication cycle in cellular compartments, known as inclusion bodies (IBs).

#### Ebola virus (EBOV)

The Ebola virus is classified into the *Filoviridae* family, whose members include another deadly virus, Marburgvirus. EBOV replication occurs in the inclusion body, where viral genomic RNA is synthesized and encapsidated by the nucleoprotein NP. The viral inclusion body was initially suggested to be a nonfunctional protein aggregate. However, recent studies have indicated their essential roles in host factor recruitment, viral RNA replication, and protein expression [155]. In the case of EBOV, inclusion bodies (IBs) lack a defined lipid membrane and contain viral proteins NP, VP35, VP40, VP30, VP24, and L [156]. The sole expression of NP or L protein, but not the other viral proteins in cells, is sufficient to induce IB formation [ 156, 157] . NP contains 751 amino acids, and the deletion of the C-terminal 98 amino acid residues of NP was found to abolish NP-induced IBs. While not required in full-length NP-induced IB formation, VP35 was reported to complement IB deficiency caused by loss of C-terminal NP, highlighting the possibility that VP35 could enhance NP-mediated condensation [157].

#### Measles virus (MeV)

The measles virus (MeV), a member of the Paramyxoviridae family, replicates its RNA in cytoplasmic inclusion bodies (IBs). Reports of MeV IBs date back to the 1950s, when these structures were noted to be globular, elongated, or irregular in the cytoplasm or the nucleus [ 158, 159] . Viral nucleoprotein (N) and phosphoprotein (P) are two key components that drive IB formation when coexpressed in cells. Both N and P are consistently detected in the IBs of infected nervous systems. These proteins harbor large intrinsic disorder domains, and IBs formed by N and P cotransfection in cells exhibit spherical morphology and undergo fusion and relaxation. FRAP assays also confirm the rapid exchange of components between IBs and the surrounding environment [160]. These condensates can recruit specific host factors for viral replication. Viral RNA could localize into N-P-mediated droplets *in vitro* and drive nucleocapsid assembly, indicating enhanced viral particle assembly by phase separation [161].

#### Rabies virus (RABV)

The neurotropic virus RABV causes fatal encephalitis in humans and animals, posing a severe threat to human healthcare. The IBs of RABV, also known as Negri bodies (NBs), were first identified in 1903 by Adelchi Negri [162]. Similar to many IBs, RABV NBs are also responsible for viral transcription and replication [163]. NBs contain all the viral replication machinery (L, N, M, and P), along with some host proteins, including HSP70 and focal adhesion kinase (FAK) [ 163, 164] . NB structures exhibit characteristics similar to LLPS-induced membrane-less condensates. Minimal coexpression of N and P proteins can reconstitute NBs, indicating the scaffolding role of both proteins in inducing NB formation [165].

#### Respiratory syncytial virus (RSV)

LLPS-induced IB formation has also been reported in respiratory syncytial virus (RSV). RSV IBs harbor viral proteins N, P, and L, as well as the viral transcription anti-terminator M2-1. The M2-1 protein can form a subcompartment within IBs, known as IB-associated granules (IBAGs) [166]. In IBAGs, viral genomic RNA and M2-1 are concentrated, but N, P, and L proteins are excluded from the structure. IBAGs undergo assembly and disassembly in cells, indicating high dynamics of these structures. Newly synthesized RNA is transiently stored in IBAGs and then transported to the cytosol, highlighting the critical role of this structure in RNA trafficking. Interestingly, the NF-κB subunit of p65 is sequestered in RSV IBs but not in IBAGs, which may facilitate viral evasion from host innate immunity [167]. Oligomerization mediated by the N protein is essential for condensation, and N and P proteins are sufficient to reconstitute the IB *in vitro* [168].

#### Vesicular stomatitis virus (VSV)

A similar example of IBs comes from the widely used model virus VSV. The replication compartments of VSV are formed by the co-expression of N, P, and L proteins, which exhibit dynamic fusion and fission [169]. The formation of this structure is not dependent on viral genomic RNA, indicating an intrinsic feature of the viral N, P, and L proteins.

Overall, IBs of negative-stranded RNA viruses have been proven to be liquid-like structures formed by transcription or replication-associated factors in the cytoplasm of infected cells. RNA viruses replicating in the nucleus, such as influenza A virus (IAV), also utilize LLPS to facilitate their propagation.

#### Influenza A virus (IAV)

Influenza A viruses belong to the Orthomyxoviridae family and contain a segmented single-stranded, negative-sense RNA. An eight-partite RNA genome is characterized, and each RNA segment is encapsidated as an individual viral ribonucleoprotein (vRNP) complex [170]. Once replicated in the nucleus, viral RNA is eventually transported into the cytoplasm as vRNPs and then to the plasma membrane. vRNPs accumulate near the microtubule organizing center (MTOC) and co-localize with the pericentriolar recycling endosome marker Rab11 [171]. These structures are membraneless and display various characteristics of liquid-like condensates. The formation of liquid-like vRNPs is suggested to facilitate viral genome packaging in the early stage of infection [172]. It is a general mechanism for viral replication machinery to be enriched in specific compartments inside host cells, enabling the efficient replication of the virus RNA and shielding the virus from host immune response recognition.

### Positive single-stranded RNA virus

Although the vRCs of positive-stranded RNA viruses are usually associated with cellular membrane systems, increasing evidence shows that proteins encoded by positive-stranded RNA viruses can also undergo phase separation and play a crucial role in the viral life cycle. For example, the SARS-CoV-2 nucleocapsid N protein has been intensively investigated, revealing its strong LLPS propensity and important function in viral genome packaging and innate immunity repression.

#### SARS-CoV-2

During SARS-CoV-2 virion assembly, the structural nucleocapsid N protein associates with viral genomic RNA and packages into RNP complexes. Approximately 40% of the N protein sequence is predicted to be intrinsically disordered [173]. Consistent with this characteristic, the N protein can undergo LLPS *in vitro* without RNA [ 140, 173– 175] . However, RNA has been demonstrated to influence N protein phase separation in a dose- and sequence-dependent manner. Specifically, a low amount of ssRNA promotes N protein condensation, while excess RNA disrupts N protein condensation *in vitro* [ 173, 176] . Meanwhile, different sequence patterns N protein viral genomic RNA play a significant role in modulating N-protein LLPS, either promoting or inhibiting protein condensation and altering the material properties of the condensate [140]. Moreover, post-translational modifications, such as phosphorylation in the serine rich (SR) region, enhance N protein LLPS and increase the fluidity of the condensate [ 174, 175] . ATP has also been shown to modulate N protein phase separation behavior [177]. High temperatures, such as 40°C, are favorable for N protein LLPS, aligning with a fever state during viral infection [140]. The N protein is reported to co-phase separate with the viral transmembrane M protein to mediate virion assembly. However, the N-M condensate is mutually excluded from the N-RNA condensate, as revealed by super-resolution microscopy, forming an outer shell-like structure enclosing the N-RNA condensate, suggesting heterogeneous organization in viral particle assembly [175].

Apart from its critical role in assembling viral particles, N protein condensation is also crucial in repressing MAVS aggregation, thus inhibiting the MAVS-dependent interferon response [178]. Disrupting N protein LLPS via a designed interference peptide alleviates immunosuppression and reduces viral titers *in vivo* [178], strongly supporting that targeting N protein LLPS could be used as an efficient strategy to restrict viral replication.

Stress granules are formed upon viral invasion and are considered one of the host’s antiviral strategies by shutting off viral mRNA translation. N protein is reported to be enriched in SGs [ 173, 175, 179– 181] . However, the outcome of N protein partitioning is controversial. Two groups claimed that SG number is reduced by N protein overexpression or SARS-CoV-2 infection after 1 hour of sodium arsenite treatment [ 179, 180] . Nevertheless, another paper concluded that N protein does not affect sodium arsenite-induced SG number after 1 hour of incubation but hinders SG disassembly [181]. However, the authors also mentioned that after more extended induction (5 h), the SG number decreased in N protein-expressing cells. Despite this divergence, it is agreed that G3BP1 and G3BP2 are two major binding partners of the N-protein [ 175, 179, 180] , which function to limit viral replication *in vivo* [179]. A detailed analysis of N protein colocalization with several SG markers revealed that G3BP1, but no other markers, such as UBAP2L, DDX1, and EIF3η, are included in cellular N protein foci [175], indicating that N protein may not simply copartition into SGs but instead actively rewire SG composition and assemble *de novo* viral granules distinct from host SGs. In addition, the fluidity of G3BP1 is reduced in N protein-invaded SGs; other SG-associated proteins, such as FUS, TDP43, and hnRNPA2, are found to form enhanced amyloid aggregation, suggesting that the N protein alters SG dynamics and promotes SG phase transition from liquid-like to solid-like [181]. Therefore, SARS-CoV-2 utilizes the N protein to target G3BP and SGs to facilitate its propagation, although a unified mechanism requires more investigation.

#### Rotavirus

In addition to SARS-CoV-2, another example of LLPS of positive-strand ssRNA virus comes from Rotavirus. The interaction between viral non-structural protein 2 (NSP2) and non-structural protein 5 (NSP5) results in spherical puncta formation in the cytoplasm, which also includes other viral factors, such as VP1, VP3, VP2, and VP6, along with its genomic RNA [182]. Mutation of S67A from NSP5 leads to aberrant spindle-like viroplasms and significantly fewer virions. Additionally, a phosphomimetic mutation of S313D in NSP2 also causes a reduction in progeny production, revealing the complex role of phosphorylation in regulating NSP2 and NSP5 condensation during viral particle assembly. Condensates formed by NSP2/5 undergo a phase transition from liquid-like to gel-like at the late stage of viral infection, and the implication remains to be further explored [77]. Rotavirus condensate can remodel SGs and p-bodies to selectively exclude some SG and p-body components while enriching others. Many SG and p-body components showed an anti-viral effect against rotavirus in cellular assays [183].

#### HIV-1

As a retrovirus, HIV-1 bears a positive-sense single-stranded RNA, which will be reverse-transcribed into a DNA molecule and integrated into the host genome. The HIV-1 nucleocapsid protein (NC) has recently been reported to phase separate in a zinc ion-dependent manner. The Zn ^2+^ chelator TPEN disrupts NC LLPS and relieves the HIV-1-induced blockade of SG [184]. Additionally, clustering of vRC of HIV-1, consisting of vRNA, reverse transcriptase (RT), and integrase (IN), was found to accumulate in nuclear speckles and facilitate integration into speckle-associated genomic DNA [185]. The HIV-1 NC condensate can function as a scaffold to enrich client viral proteins and vRNA to attain an HIV-1 core structure [186].

### DNA viruses

#### Herpes simplex virus 1 (HSV-1)

Herpes simplex virus 1 (HSV-1) is a DNA virus that primarily replicates in the nucleus. HSV-1 replication was reported to be associated with phase separation or assembly processes, but the evidence remains a matter of debate [78]. Viral DNA replication compartments can enrich polymerase II through non-specific DNA binding. The viral transcription factor ICP4, an intrinsically disordered protein (IDP), can form a liquid-like nuclear condensate [187]. HSV-1 tegument protein UL11, an RNA-binding protein involved in viral replication and cell-cell spreading, is another IDP capable of undergoing LLPS *in vitro* [188]. Further studies are needed to clarify the relevance of individual viral protein LLPS in the context of viral infection in human cells. Given that the genome of HSV-1 is approximately 150 kb and encodes approximately 80 open reading frames (ORFs), it remains to be seen to what extent LLPS plays a role in HSV-1 replication and the viral life cycle.

#### Kaposi’s sarcoma-associated herpesvirus (KSHV)

KSHV’s extrachromosomal circular DNA formation and virally encoded latency-associated nuclear antigen (LANA) can form LANA-associated nuclear bodies (LANA-NBs) during latent viral infection. Recently, LANA-NBs have been shown to form via the liquid-liquid phase separation process [189]. DAXX, a component of the PML nuclear body, is recruited into LANA-NBs during the latent stage but is evited during the transition to the lytic stage, demonstrating an example of viral remodeling of host condensates. PML exhibits a significant anti-viral effect. The interaction between LANA-NBs and PML requires further study. KSHV encodes a cGAS inhibitor, KicGAS/ORF52, which is crucial for host immune evasion. KicGAS can oligomerize and competitively bind to DNA for cGAS inhibition [ 76, 190] .

#### Epstein–Barr virus (EBV)

EBV is a strong oncogenic virus for human cells and is closely associated with various types of human cancer. As the master transcription factor and its co-factor, EBNA2 and EBNALP form nuclear condensates with liquid-like properties at the super-enhancer sites of MYC and Runx3 to activate these oncogenes [191].

### Further questions on viral condensates

The brief examples provided above, which include positive-strand and negative-strand RNA viruses as well as DNA viruses, demonstrate the prevalence of viral inclusions formed during the viral life cycle. The characteristics of these structures align with the properties of biomolecular condensates such as P granules, SGs, and p-bodies. Two areas require further study, which will not only enhance our understanding of viral intervention but also contribute to our knowledge of basic biological processes.

First, a comprehensive characterization of the compositions of each viral inclusion would provide insight into the assembly and regulatory mechanisms of these viral condensates. What host proteins are recruited into the condensate? Are biomolecules other than proteins involved, such as RNA and small metabolites? What is the relationship between viral and host condensates, such as SGs, p-bodies, and nucleolus?

Second, how does the condensation of viral and host biomolecules facilitate viral genome replication and protein expression? How does the viral condensate evade the host immune system, and what roles do these condensates play in viral-host interactions? Given the broad suggestion in the literature of the anti-viral effect of SG, further studies focusing on the viral condensate and human SG could facilitate the discovery of the roles condensates play in this arms race interplay and may lead to the development of novel therapeutic interventions.

## The Interplay between Virus and Host Stress Granules

**Figure 3:**
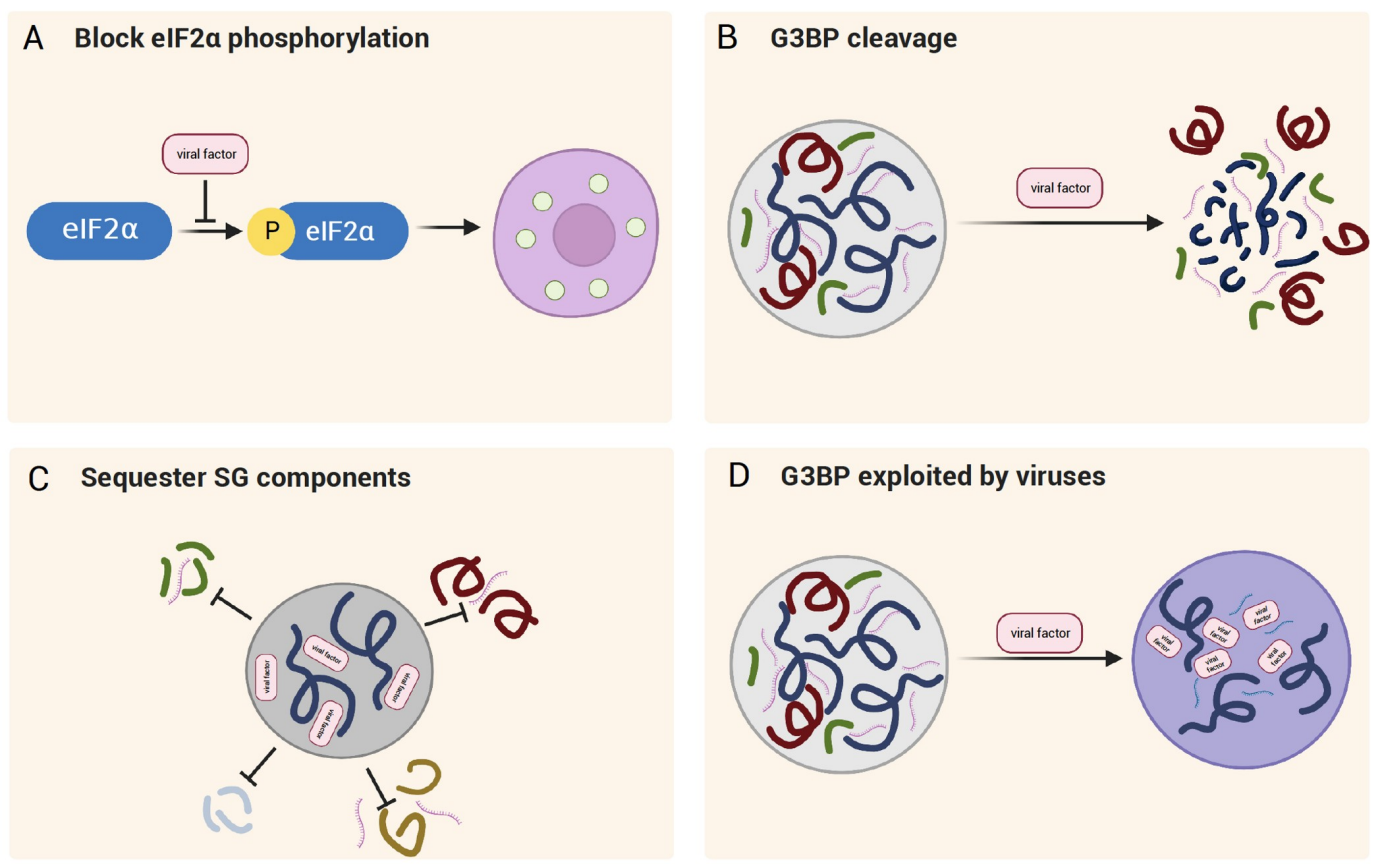
Figure 3 Viral-host interactions via condensate modulation Multiple pathways are used in viral-host interactions and affect the host condensate differently. Examples are signaling intervention via eIF2α (A), host protein cleavage (B), and hijacking (C,D).

In the following sections, we will discuss viral remodeling in eIF2α signaling and interactions with SG proteins. The assembly of SGs upon viral infection is multifaceted, with many examples in the literature reporting the inhibition of SGs during viral replication. However, caution is necessary, as many studies use only one or a few SG markers. Whether the canonical SG is formed or a specific SG protein is recruited to the structures is typically not examined. Collective studies suggest an antiviral function of SGs during certain stages of viral infection. Viral protein synthesis is strictly dependent on host translational machinery. Bulk translational shut-off alongside SG formation significantly hinders efficient viral propagation. Consistent with this assumption, there is substantial evidence that viruses have evolved various strategies to antagonize SG ( Figure 3).

### Repression of eIF2α phosphorylation and prevention of SG assembly

Given the absolute dependence on host translational machinery for viral protein production, the host defense system is rapidly mobilized to arrest translation upon sensing viral pathogen-associated molecular patterns (PAMPs) [192]. Translation initiation in mammalian cells is regulated by the eIF4F complex, which consists of the cap-binding protein eIF4E, the RNA helicase eIF4A, and the scaffold protein eIF4G. The eIF4F complex recruits the 43S complex, which consists of a 40S ribosomal subunit bound to the ternary complex of eIF2-GTP/Met-tRNAi, eIF1A, eIF1, and eIF3. Poly(A)-binding protein (PABP) binds to the eIF4F complex and the polyadenylated tail of the mRNA, forming the 43S pre-initiation complex (PIC) that scans the mRNA from 5′ to 3′ to recognize the AUG start codon [193]. Additional proteins, including eIF1, eIF1A, eIF2, and eIF5, form the 48S complex which is delivered to the ribosome for translation initiation mediated by a complex composed of eIF2, GTP, and the initiator methionyl tRNA (Met-tRNA) [194]. The eIF2-GTP-Met-tRNA ^Met^ ternary complex binds with the 40S subunit in a GTP-dependent manner. During translation initiation, inactive eIF2-GDP is recycled back into the active form (eIF2-GTP) by eIF2B [195]. The stalling of translation under stress conditions is primarily regulated by the phosphorylation of eIF2α at serine 51. Phosphorylated eIF2α results in the sequestration of eIF2B from binding to eIF2, leading to the disassembly of polysomes, translation arrest, and the formation of stress granules [196].

Phosphorylation of eIF2α can be catalyzed by one of four eIF2α serine/threonine kinases: PKR, PERK, HRI, and GCN2. To antagonize host translation inhibition induced by eIF2α phosphorylation, viral proteins typically target either the four kinases or eIF2α itself. Newly translated viral proteins can bind to viral RNA, thus protecting it from PKR recognition and inhibiting PKR activation. Examples include the SARS-CoV -2 N protein [197], VACV E3L protein [198], human cytomegalovirus (HMCV) TRS-1 and IRS-1 proteins [ 199, 200] , reovirus σ3 [201], HSV-1 Us11 [202], Middle East respiratory syndrome coronavirus (MERS-CoV) accessory protein 4a [ 203, 204] , NS1 from influenza A virus (IAV) [ 205, 206] , and VP35 of Ebola virus (EBOV) [ 207, 208] . PKR dimerization, which is required for activation, is targeted by several viral proteins, such as HCV NS5A protein [209] and Japanese encephalitis virus (JEV) NS2A protein [210]. In addition, adenoviral RNA-I (VAI), a short noncoding RNA, prevents dimerization by binding with one of the monomers, thus inactivating PKR [211]. Another strategy to evade PKR surveillance is the self-restriction of dsRNA ligands. The conserved endoribonuclease nsp15 in different genera of coronaviruses has been shown to reduce viral dsRNA accumulation, thereby preventing PKR activation and SG formation [212].

In addition to targeting upstream kinases, the phosphorylation of eIF2α can also be directly affected by certain viruses. The nucleoprotein (N) and glycoprotein precursor (GPC) of Junín virus (JUNV) impair the phosphorylation of eIF2α [213]. HCV utilizes host protein phosphatase 1 (PP1) and its regulatory subunit GADD34 (growth arrest and DNA-damage-inducible 34) to de-phosphorylate eIF2α [ 214– 216] . AcP10 of the human gastroenteric picornavirus Aichivirus was reported to bind with eIF2B to inhibit eIF2B sequestration, maintaining the interaction between eIF2B and eIF2 to continue translation initiation [217].

### G3BP1 and other SG proteins are targeted to disrupt SG

SGs serve as crucial antiviral hubs for hosts. Therefore, direct targeting of core SG proteins such as G3BP is a common and straightforward way to manipulate the host antiviral response. SG assembly occurs at the early stage of poliovirus (PV) infection, but the viral 3C protein cleaves G3BP at the later stage, leading to SG disassembly [218]. Similar strategies are seen in foot-and-mouth disease virus (FMDV) [ 219, 220] , encephalomyocarditis virus (EMCV) [206], coxsackievirus B3 (CBV3) [221], Enterovirus71 (EV71) 3C [222] and feline calicivirus (FCV) NS6(Pro) [223], where G3BP proteins are targeted and cleaved.

In addition to G3BP, several other SG proteins are degraded to disrupt antiviral SGs. The 2A protease of picornaviruses (EV71, PV, CVA) induces what is termed atypical SG by cleaving eIF4GI and selectively sequestering host mRNA, leaving viral mRNA intact and thereby facilitating viral translation [224]. Notably, the induced atypical SG does not contain G3BP1 and some eIFs [224]. Using a similar cleavage strategy, HIV-1, HIV-2, SIV, mouse mammary tumor virus (MMTV), and Moloney murine leukemia virus (MoMLV) inhibit SGs by cleaving eIF4GI and eIF4GII. HIV-1, HIV-2, and MMTV also cleave PABP to arrest host cap-dependent mRNA translation while leaving viral translation unaffected [225].

The cleavage sites of G3BP are located before the RRM and RGG motifs. The cleaved fragments are defective in RNA binding and in mediating LLPS with RNA, as indicated in cellular and *in vitro* reconstitution experiments. One unresolved question is whether the effect on viral replication occurs through the G3BP protein or the mesoscale SG formed by G3BP. Future interventions aimed at disrupting SGs without affecting the integrity of the G3BP protein could help answer this question.

### Sequestration of SG components to disable SG

Apart from the cleavage of G3BP and related RBPs, viruses also employ strategies to disrupt SGs by sequestering components such as G3BP, Caprin1, and TIA-1. SARS-CoV-2 N protein inhibitspoly(I:C)-induced antiviral SGs by interacting with G3BP1 [ 197, 226, 227] . This interaction leads to the rewiring of host mRNA binding profiles and alters stress gene expression [227]. Dengue virus (DENV) and West Nile virus (WNV) trigger the relocation of the SG protein TIA-1/TIAR to viral replication sites, thereby interfering with SG formation [97]. Zika virus (ZIKV) sequesters the core SG proteins G3BP1, TIAR, and Caprin-1, facilitating viral replication and causing SG disassembly [228]. Japanese encephalitis virus (JeV) co-opts Caprin-1 to disrupt SGs [ 229, 230] , while Sendai virus (SeV) uses its trailer RNA to trap TIAR from SGs [231]. The leader protein of Theiler’s Virus (TMEV) inhibits SG formation on two fronts: it blocks the interaction between PKR and viral dsRNA and sequesters G3BP1 without affecting eIF3 [ 232, 233] . HTLV-1 represses SG formation by interacting with histone deacetylase 6 (HDAC6) [234]. Hepatitis C virus (HCV) infection also involves hijacking SG components and redistributing G3BP1, ATXN2, DDX3X, and PABP1 to the viral replication site near lipid droplets [ 235, 236] . These examples illustrate the overall effect of viral interaction with RNA-binding proteins, especially RBPs, in SGs. Further investigation of virus-remodeled structures and comparisons with canonical SGs induced by SA or heat shock will reveal the extent of viral modeling of the SG proteome. The recent *in vitro* reconstitution of SG also provides a platform to systematically investigate the effect of virus-encoded proteins on SG assembly.

### Turning an opponent into an ally: viral manipulation of G3BP from antiviral to proviral

While G3BP1 has an antiviral effect in stress granules, it is now clear that direct degradation or inhibition of G3BP does not always benefit viral replication. Several viruses have evolved a sophisticated strategy to inhibit SGs and coopt G3BP1 for their own replication. A prime example is the alphavirus, a genus of approximately 30 members. Old-world alphaviruses, such as Chikungunya virus (CHIKV), Semliki Forest virus (SFV), and Sindbis virus, bind to G3BP via their nsP3 protein through an FGxF motif, mimicking the host protein Caprin-1/USP10 [ 237– 245] . New-world alphaviruses, such as Eastern, Western, and Venezuelan equine encephalitis virus (EEEV, WEEV, and VEEV) nsP3s, sequester the SG protein FXR1/FXR2/FMR1. Despite the difference in interaction, deletion of the respective binding partners is detrimental to the assembly of the alphavirus replication complex [ 240, 246, 247] .

Similar observations can be found in murine norovirus (MNV) infection, where the virus rewires the G3BP1 interactome to activate metabolic reprogramming without inducing SG. Consequently, silencing of *G3BP1* impairs MNV replication, similar to what has been reported for alphaviruses [ 248, 249] . Human norovirus has also been reported to rely on G3BP for its replication. G3BP1 was identified as an essential factor of both human and murine noroviruses in an unbiased CRISPR-Cas9 screening. In G3BP-deficient cells, VPg protein-dependent viral translation is significantly reduced. Moreover, G3BP affects viral replication before the viral negative-strand synthesis step, suggesting that G3BP likely acts as a critical factor at the very early and essential stage of viral invasion [250]. In addition to the “arms race mode” of host and virus interaction, the proviral role of G3BP/FXR in alphavirus/norovirus possibly represents a more complex “domestication mode” of host proteins by viruses.

## Concluding Remarks and Perspectives

Viral proteins can disrupt host cell condensates to facilitate replication and inhibit the host innate immune responses. SGs and p-bodies are prevalent targets due to their crucial role in RNA metabolism, including RNA translation and stability. SARS-CoV-2 can inhibit SG formation via its nuclear capsid protein N [ 179, 197, 226, 227] . SARS-CoV-2 can also abolish p-body formation. However, no other coronaviruses have been found to disrupt p-bodies that inhibit cytokine mRNA translation inside the p-body. This unique ability may contribute to the cytokine storm seen in COVID-19 patients [251]. SGs have been shown to buffer the release of dsRNA into the cytosol for innate immune activation [252] and prevent an excessive innate immune response upon dsRNA stimulation [253]. Further investigation of the specific effects of SARS-CoV-2 and other viruses on SGs and p-bodies may provide a unique angle for antiviral targeting.

The context of SG research can further provide insight into the physiological and pathological functions of proteins related to SGs, which have traditionally been studied in model systems. The interaction between SGs and viruses provides a frontier in the evolutionary selection process of viral infection. Research over the past decades has revealed many phase separation-related organelles formed by viral proteins, and many host MLOs are remodeled by viral infection. The intricate connection to innate immunity provides another important angle to study the role of SGs and related MLOs in viral-host interactions.
